# A Rare Case of Deep Digital Flexor Tendinopathy following Centesis of the Navicular Bursa

**DOI:** 10.3389/fvets.2017.00169

**Published:** 2017-10-16

**Authors:** Tim J. Froydenlund, Lucinda J. Meehan, Linda R. Morrison, Raphael Labens

**Affiliations:** ^1^Fyrnwy Equine Group, Shrewsbury, United Kingdom; ^2^Diagnostic Imaging, School of Veterinary Sciences, University of Bristol, Langford, United Kingdom; ^3^Easter Bush Pathology, Royal (Dick) School of Veterinary Studies, The Roslin Institute, The University of Edinburgh, Midlothian, United Kingdom; ^4^Faculty of Science, School of Animal and Veterinary Sciences, Charles Sturt University, Wagga Wagga, NSW, Australia

**Keywords:** horse, navicular bursa, centesis, deep digital flexor tendinopathy, foot

## Abstract

Navicular bursa (NB) centesis is a common diagnostic and therapeutic procedure in equine practice. This case report documents the clinical, diagnostic imaging and histological findings in a horse with a suspected iatrogenic deep digital flexor tendon (DDFT) injury following centesis of the NB *via* a modified distal plantar approach (placement of two needles in a weight bearing position). Although it cannot be proven with absolute certainty, the authors believe that this is the first reported case where NB centesis is the likely cause of a DDFT lesion, and with magnetic resonance imaging performed both pre- and post-centesis. With this potential, though rare, complication of the procedure, alternative tendon sparing injection techniques should be considered prior to NB centesis in certain cases.

## Introduction

Centesis of the navicular (or podotrochlear) bursa (NB) is regularly required for diagnostic or therapeutic purposes in the management of foot lameness. A palmar approach is most commonly used (1). Schramme et al. (2) compared five different techniques and found that the distal palmar approach to the “navicular position” allowed accurate and reliable centesis, regardless of foot conformation. This finding is supported by others who have found this approach to be more successful than when a needle is advanced parallel to the solar surface (3). Radiography is generally recommended to ensure accurate needle placement, minimising the number of needle passes through the deep digital flexor tendon (DDFT) and optimising fluid retrieval from the bursa (1, 3, 4). Concern remains regarding iatrogenic injury to the DDFT with bursocentesis, although to date this has not been reported (4). Horses typically undergo repeat bursocentesis for the purpose of corticosteroid administration. This has been associated with spontaneous rupture of the DDFT (5) and similar complications are known to occur in people (6, 7). However, the effect of the procedure versus that of the corticosteroid is impossible to differentiate and given the lack of advanced diagnostic imaging in the only related equine report (5) pre-existing DDF tendinopathy may contribute to this complication. Nevertheless, to minimise the potential for iatrogenic damage a lateral or DDFT sparing approach to the NB has recently been reported and may be preferred (8). On the basis of the following case report and under the conditions specific to this case, we present the possibility of clinically significant iatrogenic DDFT injury, following a palmar/plantar approach to the NB.

## Background

A 5-year-old Warmblood gelding was referred to the Royal (Dick) School of Veterinary Studies for evaluation and treatment of severe right hindlimb (RH) lameness, following a solar puncture 19 days previously. The lameness had reportedly transiently improved following a 5-day course of oral potentiated sulphonamides. Prior to the onset of lameness, there was no pre-existing history of lameness.

## Case Presentation

### Clinical Examination

Clinical examination found the right hind digital pulses to be bounding and the horse was toe-touching lame on the RH at the walk (grade 5/5 AAEP). On examination of the foot, a small tract within the lateral paracuneal sulcus was seen, located midway along the frog in a dorsoplantar direction. There was a small volume of purulent discharge from the solar defect and from a further tract adjacent to the lateral heel bulb.

### Radiography

**Figure 1:**
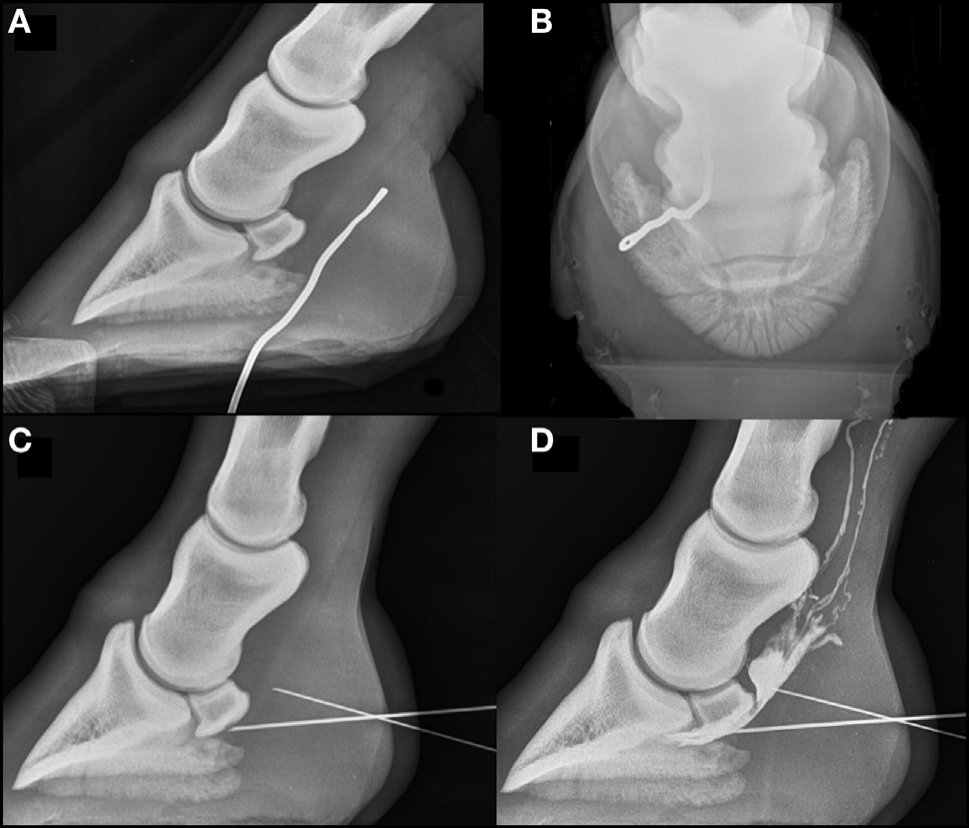
Radiographs of the right hind foot acquired on the day of admission. Dorsal is to the left on lateromedial images and lateral to the left on dorsoproximal images. **(A,B)** Lateromedial and dorsal60proximal–plantarodistal oblique views of the right hind foot with a metal probe in place in the discharging tract. The probe can be seen passing just lateral to the midline in a plantaroproximal direction to the level of the proximal third of the middle phalanx. No osseous abnormalities are seen. **(C)** Standing lateromedial view with a needle in place within the navicular bursa (NB), contacting the plantar compact bone of the navicular bone. There is a second needle further proximally, at the plantar extent of the deep digital flexor tendon (DDFT), which is the needle subsequently inserted into the proximal recess of the NB. **(D)** Positive contrast bursogram showing that the NB is intact. Both needles described in **(C)** remain in place. A small volume of contrast is extravasated plantar to the DDFT and lymphatic drainage of contrast is seen proximally.

On radiography (lateromedial and dorso 65° proximal-plantarodistal oblique views) with a blunt metallic probe inserted into the defect (Figures 1A,B), the tract was seen to extend approximately 8 cm in a proximoplantar and axial direction in close proximity to the plantar aspect of the DDFT and the distal extremity of the digital flexor tendon sheath (DFTS, Figure 1). Radiographic artifaects associated with presence of a sub-solar foot abscess were not appreciated.

### Synoviocentesis

Synoviocentesis of the RH distal interphalangeal joint (DIPJ), using a dorsal perpendicular approach (9), yielded a synovial fluid sample, which was grossly and cytologically normal. Due to the infected tissue on the plantar aspect of the digit and a proximally discharging sub-solar abscess, synoviocentesis of the NB was not performed at this stage.

### Magnetic Resonance Imaging

The RH foot was examined using a low-field (0.27-T) open system designed for use in standing horses (Hallmarq EQ 2; Hallmarq Veterinary Imaging Ltd., Guildford, Surrey, UK). The following protocol was used: T1-weighted (T1W) 3D gradient echo (GRE), T2*-weighted (T2*W) 3DGRE, T2 weighted (T2W) fast spin echo (FSE), and short tau inversion recovery FSE sequences in multiple orthogonal planes with slice thicknesses between 1.5 and 5 mm.

**Figure 2:**
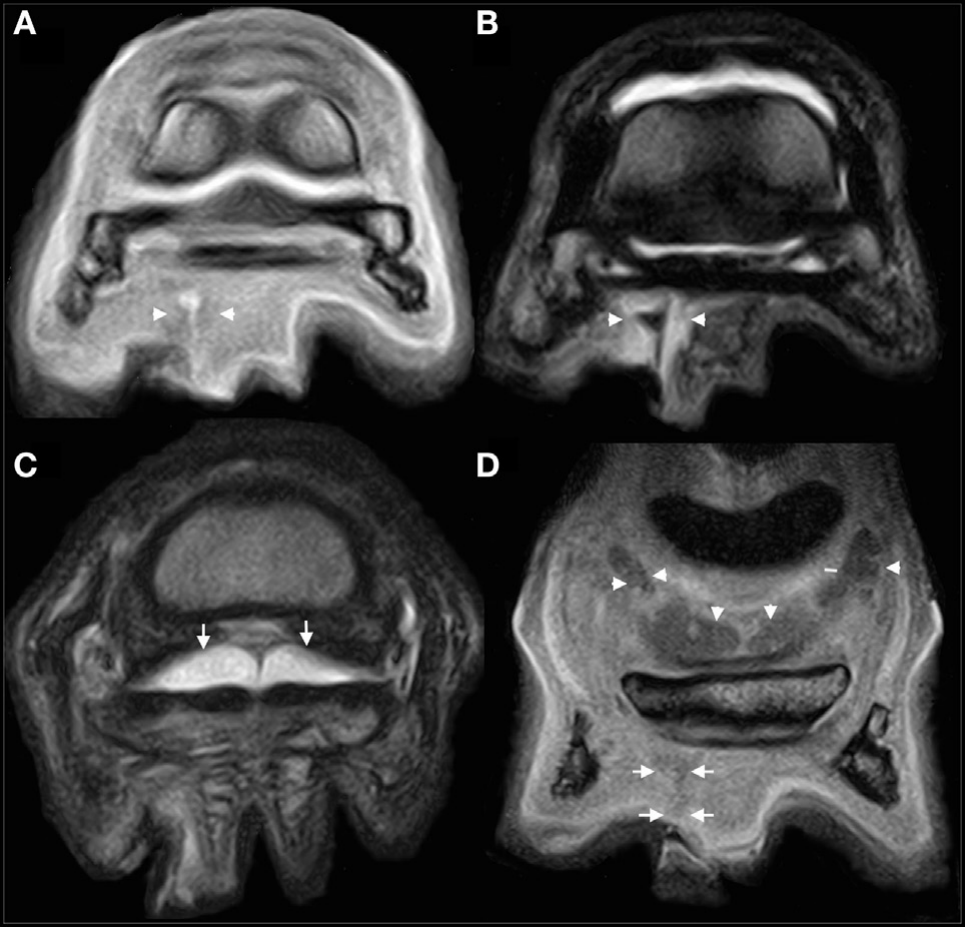
MRI images of the right hind foot acquired at the time of presentation. Lateral is to the left of all images. **(A,B)** Slice matched transverse T1-weighted (T1W) gradient echo (GRE) **(A)** and T2-weighted (T2W) fast spin echo (FSE) **(B)** images at the level of the distal horizontal border of the navicular bone. The tract (arrow heads) can be seen as a hyperintense [in **(A)**] and hypointense [in **(B)**] tract extending from the lateral sulcus of the frog into the sub-solar tissues in both images. **(C)** Transverse T2W FSE image just proximal to the navicular bone within the proximal recess of the navicular bursa (NB). The bursa is seen to have marked effusion (arrows). There are no abnormalities associated with the tendon at this time. **(D)** T1W GRE frontal image at the plantar extent of the tract. The tract can be seen as an ill-defined hypointense tract just proximal to the lateral sulcus of the frog (arrow). Marked effusion of the NB can be appreciated (arrowheads).

**Figure 3:**
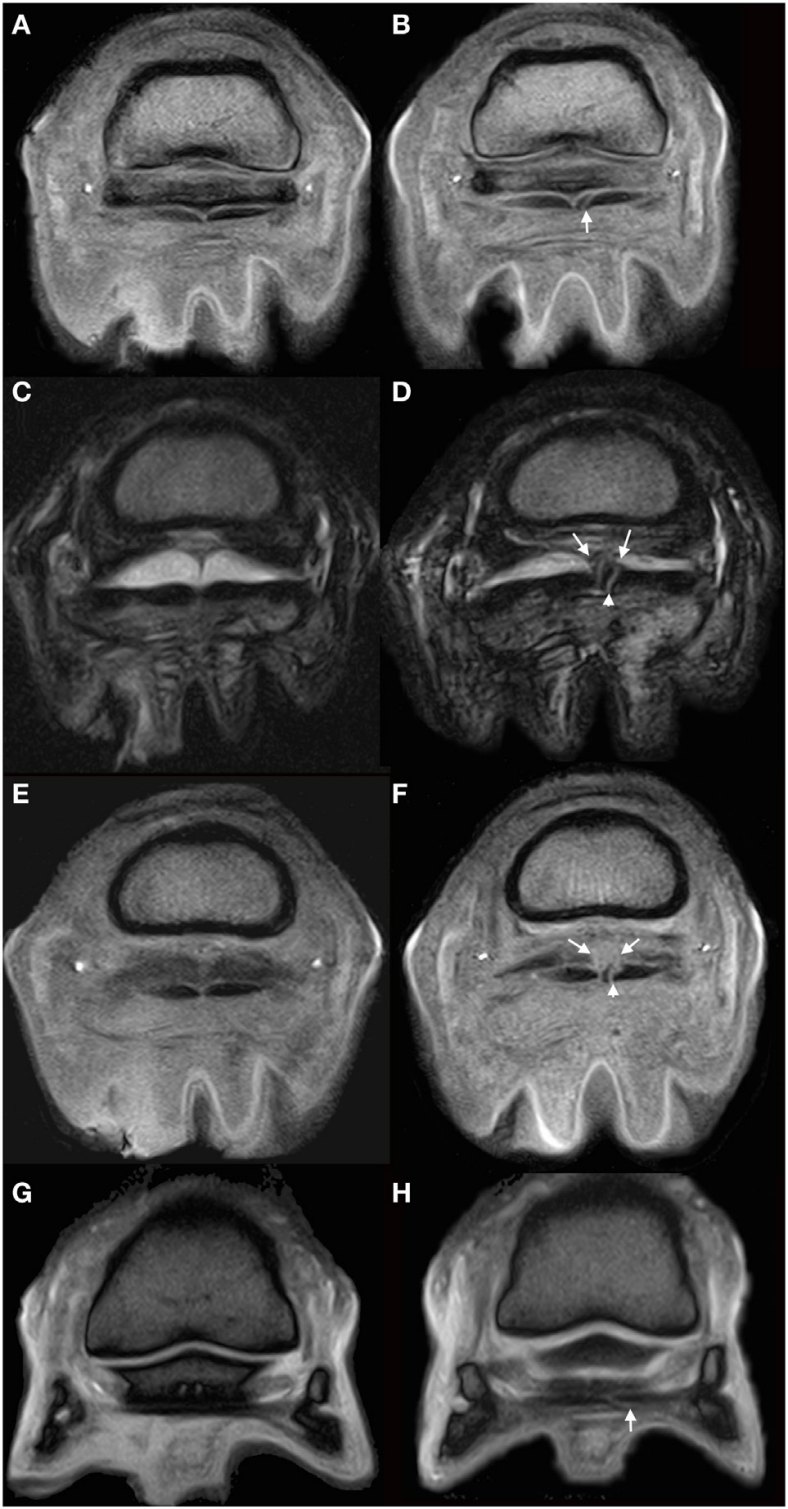
MRI images of the right hind foot. Lateral is to the left of the images. **(A,B)** Slice matched T1-weighted gradient echo (GRE) images of the right hind foot at the level of the proximal third of the navicular bone. **(A)** was acquired at the time of admission and **(B)** at the time of representation. In panel **(A)**, the medial lobe of the deep digital flexor tendon (DDFT) is normal in appearance. In panel **(B)** a well-defined hyperintense line is seen traversing the axial portion of the medial lobe from plantar to dorsal, consistent with a parasagittal split in the tendon (white arrow). **(C,D)** Slice matched T2-weighted fast spin echo images within the proximal recess of the navicular bursa (NB), acquired at initial presentation **(C)** and the time of repeat presentation **(D)**. In image **(D)**, the previously described hyperintense linear lesion in the medial lobe is seen (arrowhead). There is well defined linear material extending dorsally from the medial portion of the DDFT to the collateral sesamoidean ligament (arrows), which is markedly increased when compared to that seen in **(C)**. The NB remains effused. **(E,F)** Transverse slice matched T1GRE images within the proximal recess of the NB acquired at initial presentation **(E)** and repeat presentation **(F)**. The hyperintense linear lesion within the medial lobe is clearly seen in panel **(F)** (arrow head). There is a region of mildly hyperintense material dorsal to the tendon in panel **(F)** (arrow), which is consistent with the presence of adhesions as seen in image **(D)**. **(G,H)** Slice matched transverse T2*WGRE image at the distal horizontal border of the navicular bone. The previously described lesion in the medial lobe of the DDFT remains visible in panel **(H)** at this level (arrow) demonstrating continuation of the lesion distal to the navicular bone.

A wide tract of hypointense signal surrounding a markedly hyperintense region (GRE sequences) and markedly hyperintense signal surrounding a hypointense region (T2FSE sequences) was seen to extend dorsoproximally from the lateral sulcus of the frog towards the plantar border of the DDFT at the level of the distal third of the navicular bone. This was interpreted as consistent with a tract containing gas or haemosiderin, represented by the hypointense regions and proteinaceous fluid material (Figures 2A,B) The DDFT appeared normal in all sequence types throughout the imaged field (Figures 2 and 3A). The NB was markedly distended (Figures 2C,D) possibly consistent with NB sepsis and so, despite the adjacent abscess, synoviocentesis was considered prudent. Systemic and regional antimicrobial therapy had been commenced the previous day and so the risk of iatrogenic infection post-centesis was considered reduced.

### NB Centesis

Following local perineural analgesia of the plantar nerves at the level of the base of the proximal sesamoid bones and under radiographic guidance, a lateral approach to the NB was attempted, but no synovial fluid sample could be obtained. Synoviocentesis was then attempted by the distal plantar approach to the “navicular position” with the horse in a weight bearing position (2). The patient had not been compliant with a previous attempt to perform centesis in a non-weight-bearing position using a Hickman block. Despite the needle being placed in the correct position (Figure 1C) fluid could not be aspirated. A second needle was placed in the proximal recess, resulting in two needle advancements through the DDFT (Figures 1C,D). The first needle was left *in situ* and used as a guide during placement of the second needle. This yielded a turbid, pale synovial fluid sample with a total nucleated cell count of 2.04 × 10^9^/L and total protein of 62 g/l consistent with synovial inflammation but not sepsis (10, 11). Subsequent positive contrast bursography, using 4 ml iohexol (Omnipaque; GE Healthcare Ireland, Cork, Ireland), confirmed the integrity of the NB. The duration of needle placement was kept to a minimum and only that time required for confirmatory radiography, collection of a synovial sample, distension of the bursa with contrast material and, finally, serial radiography. The procedure was well tolerated, with the limb held stationary throughout centesis.

### Treatment

Standing debridement of the sub-solar corium and lavage of the penetrating tract was performed. A parenteral course of procaine penicillin (Depocillin; MSD Animal Health, Milton Keynes, Buckinghamshire, UK) at a dose of 22,000 IU/kg and gentamicin (Genta-Equine; Dechra Veterinary Products Ltd., Shrewsbury, Shropshire, UK) at a dose of 6.6 mg/kg was commenced and continued throughout the 4-day period of hospitalisation. This regimen was supported by three intravenous regional perfusions (IVRP) each with 500 mg amikacin (Amikin; Bristol-Myers Squibb Holdings Ltd., Uxbridge, Middlesex, UK), diluted in a total perfusate volume of 100 ml, repeated every 24 h. Each IVRP was performed *via* the medial saphenous vein with an Esmarch bandage placed as a tourniquet at the level of the mid-distal crus. Five hundred milligrams of flunixin meglumine (Flunixin; Norbrook Laboratories Ltd., Newry, County Down, UK) were administered i.v. b.i.d. for 2 days, then 1 g phenylbutazone (Equipalazone) (see text footnote 4) i.v. s.i.d. for 2 days. Two days after admission, a glue-on shoe and hospital plate were fitted by the horse’s farrier, and the sole packed with dry gauze swabs. The patient’s comfort improved steadily throughout the period of hospitalisation. At discharge and still under the effect of 1 g phenylbutazone i.v. s.i.d. the horse was sound at the walk. Discharge instructions advised box rest until the hospital plate was removed and the solar defect had keratinised fully.

### Further History

The horse represented to the hospital 147 days following discharge for re-evaluation of RH lameness. After the solar defect had fully keratinised, the horse had been on walking exercise for 6 weeks, with occasional trot and canter allowed in an indoor school. No lameness was observed by the client during this period, but veterinary examination was not performed. Paddock turnout was then reintroduced, and an equivocal RH limb lameness noted. Paddock turnout was suspended but lameness persisted.

### Clinical Examination

Clinical examination revealed widening of the RH lateral heel bulb and moderate atrophy of the right gluteal musculature. Application of hoof testers was negative. Dynamic assessment found the horse to be 4/10 RH lame (grade 3/5 AAEP) at trot in a straight line on hard surface, increasing to 6/10 (grade 3/5 AAEP) when trotting on an incline. Flexion tests of both hindlimbs were negative. Toe elevation (coffin joint extension test) in the RH resulted in moderate exacerbation of the lameness.

### Diagnostic Local Anaesthesia

Diagnostic local anaesthesia of the RH DIPJ did not alter the degree of lameness. The lameness was also unchanged following a plantar digital nerve block. A basisesamoid nerve block resulted in approximately 50% improvement in the degree of lameness, which was completely abolished following perineural local anaesthesia of the dorsal and plantar metatarsal nerves and the plantar nerves (“low 6-point” nerve block). Once the effects of the previous local anaesthesia had abated, and in order to further localise the source of pain, diagnostic local anaesthesia of the RH DFTS improved the lameness to grade 2/10 (grade 1/5 AAEP), with additional improvement after perineural anaesthesia of the medial and lateral plantar nerves alone. Local analgesia of the right metatarsophalangeal joint did not alter the level of lameness.

### Magnetic Resonance Imaging

Repeat MRI of the foot and pastern was performed using the protocol and equipment previously described. The medial lobe of the DDFT was abnormal from the mid-level of the proximal phalanx to the insertion. There was a discrete core lesion within the tendon at the mid point of the proximal phalanx, which widened into a parasagittal split at the level of the proximal recess of the NB and extended distally to involve the insertion (Figure 3). The parasagittal split was located at the axial aspect of the medial lobe of the DDFT, adjacent to the midline septum (Figures 3A–F). The lesion was seen as hyperintense on T1W and T2*W GRE sequences as it traversed the navicular bone, with focal regions of signal hyperintensity on T2W FSE sequences. There was a marked irregularity of the dorsal border of the DDFT within the proximal recess of the NB, with soft tissue material extending between the dorsal border of the DDFT and the plantar border of the collateral sesamoidean ligament, suggestive of intrabursal adhesion formation (Figures 3C,D).

### Postmortem

Following discussion with the client and in consideration of the prognosis, the decision to euthanase was made. A postmortem examination revealed a full thickness parasagittal tear in the medial lobe of the DDFT extending approximately from the level of the proximal recess of the NB to the level of the coronary band, which corresponds precisely to the proximal and distal needle entry points used during the initial work up.

### Histopathology

**Figure 4:**
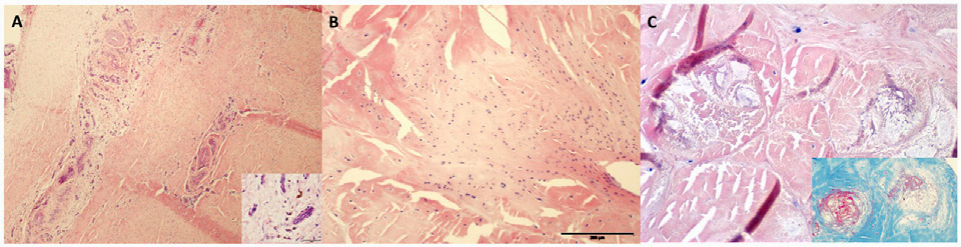
**(A)** Deep digital flexor tendon neovascularisation and fibrosis (4× magnification) with scattered haemosiderophages (inset at 40× magnification). H&E stain. **(B)** Areas of fibrocartilaginous metaplasia at 10× magnification. **(C)** Collagenolysis on H&E section of tendon at 20× magnification with inset highlighting degenerate collagen (stained red on Masson’s Trichome).

Three pieces of digital flexor tendon were taken at the time of postmortem and fixed in 10% neutral buffered formalin. Each piece of tissue was bisected and embedded in paraffin-wax; 5-µm sections were stained with haematoxylin and eosin and one section was stained with Masson’s trichrome. Histologically in all sections, there was neovascularisation with tunica media hypertrophy, multifocal to coalescing fibrosis (Figure 4A), fibrocartilaginous metaplasia (Figure 4B) and collagenolysis (Figure 4C). These changes have all been described in naturally occurring DDFT injury (12–15). Though these findings are not pathognomic for an injury caused by synoviocentesis they do not preclude it and our interpretation that these changes are related to the synoviocentesis is based on the clinical history and imaging results.

## Discussion

After the first examination, the clinical and diagnostic imaging findings in this case were consistent with a sub-solar abscess, resulting from a previous solar penetration. Advanced diagnostic imaging and a thorough clinical investigation were required to exclude the involvement of vital structures such as the DDFT and NB. In view of this diagnosis, and the patient’s response to intensive treatment, the prognosis was initially considered excellent. However, re-examination approximately 4 months later when ridden exercise had just been reintroduced, identified the presence of significant DDFT injury. In the context of the horse’s clinical history and based on the following considerations, the authors have come to suspect that the observed DDFT injury could be related to the previously performed NB centesis:

- Initial MRI examination did not reveal any abnormal findings. In contrast to high-field MRI small focal DDFT lesions (approximately 1 mm diameter) may not be detected utilising low-field MR systems (16). Given that a low-field system was used, small underlying tendon lesions may have gone undetected due to limitations in resolution. Kinns and Mair (17) reported a case series in which one horse did not have appreciable tendon damage on MRI examination within 48 h of a penetrating injury; however, when imaged 14 days following the initial insult, a tendon injury was visible on low-field MRI. The authors believe that the time elapsed between injury and imaging in this case (19 days) and the marked improvements in image quality and resolution in the MRI system over the 10 years since Kinns and Mair published their case report greatly reduces the likelihood of a lesion being missed at the initial MRI examination.
- Synoviocentesis and a contrast bursogram did not support the premise of an injury penetrating the DDFT and the NB, though this does not preclude DDFT penetration alone. The passage of the blunt metallic probe into the solar tract, subsequent radiograph and MRI examination were not consistent with a penetrating DDFT injury.
- Repeat MRI examination determined that the parasagittal tear was precisely located between the proximal and distal needle tracts through the DDFT.
- The lack of opportunity to incur such an injury in a young horse which had not previously received intrabursal corticosteroid. The horse had been ridden little and certainly not to its previous level of athleticism.
- Taken together, the very low prevalence of primary hind limb DDFT lesions [1/38 horses (18)] and the specific injury pattern (complete parasagittal split at the level of the navicular bone extending both proximally and distally as an incomplete tear or core lesion) make this an unusual lesion. Primary DDFT lesions normally extend proximally or distally from the navicular bone (19) but rarely in both directions, as seen here.

The authors believe that the DDFT tear has been caused by propagation in a proximal and distal direction from the disrupted tendon architecture at the centesis sites. This process could have occurred at any point following centesis, presumably as a consequence of limb loading. It is, however, unlikely that the entirety of the tear arose acutely at the time of centesis, but that a local inflammatory process first ensued. This inflammation, in combination with the mechanical disruption, would have consequently weakened the tendon, allowing tear propagation.

The authors also acknowledge that in this case there was an inflammatory focus in close proximity to the tendon due to the presence of the sub-solar foot abscess. Therefore, the possibility remains that with this a greater risk for subsequent DDFT injury existed. However, while this cannot be refuted with absolute certainty, clinical experience strongly suggests that sub-solar foot abscessation is not a predisposing factor for DDF tendinopathy.

The response to local anaesthesia can be explained by the assumption that a portion of the DDFT within the foot and distal to the DFTS receives its sensory supply from more proximal deep branches of the medial and lateral palmar (or plantar) digital nerves that enter the DFTS (20). These branches may be unaffected by plantar nerve blocks performed at the base of the proximal sesamoid bones. In addition, the DDFT tear extended into the intrathecal section of the tendon and it is well documented that injuries in this location often respond partially to local anaesthesia of the DFTS with soundness only achieved following perineural anaesthesia of the plantar nerves (21, 22). The presence of a pre-existing DDFT injury within the DFTS cannot be excluded, since magnetic resonance imaging of the foot alone was performed during the first examination thereby only visualising the most distal portion of the DFTS. The absence of pre-existing lameness or DFTS distension, however, renders a pre-existing tendon injury an unlikely possibility.

Comparatively, little is known regarding the aetiology of DDF tendonopathy in the horse (23). Primary DDF tendonitis may be the result of repetitive overuse or an acute-onset traumatic insult, possibly superimposed on pre-existing degenerative change (19). The identification of severe core lesions in young horses that have done relatively little work suggests that some horses may have an inherent predisposition to injury (19). Age and accumulation of degenerative ageing changes in the tendon may also have an effect on the risk of primary tendon injury (12, 13). Considering the age of the horse, the nature of the injury and the absence of significant tendon abnormalities on the previous MRI scan, prior degenerative disease of the tendon seems unlikely in this case.

Labens and Redding (4) discuss the dilemma of whether to inject the NB, suggesting that, in horses with suspected DDFT injuries, one might prefer to avoid repeat bursal injections and consequent penetration of the DDFT so that an already weakened structure is not further traumatised. The role of needle placement and/or the potential degenerative effect of intrabursal corticosteroids in DDFT weakness remain unknown. Placement of two needles in a weight-bearing position with the tendon under tension may also have facilitated this injury albeit this suspicion cannot be substantiated. Nevertheless, in the context of this case, we would suggest that one should aim to minimise the number of needle advancements through the DDFT using radiographic guidance. A radiographic guided injection technique which avoids penetration of the DDFT by approaching centesis from the lateral aspect of the limb would represent the best option (8).

## Concluding Remarks

In summary, although it cannot be proven with absolute certainty, the authors believe that this is the first reported case where NB centesis is the likely cause of a DDFT lesion and with MRI scans performed both pre- and post-centesis. Although it is an unusual complication of the procedure, the authors believe is should be recognised as a potential sequela. The distal palmar/plantar approach remains a practical and reliable procedure but alternative tendon sparing techniques should be considered, particularly in cases with suspected DDFT injury or, indeed, in cases with adjacent sub-solar abscessation. Placement of two needles through the tendon simultaneously should be avoided.

## Ethics Statement

The horse detailed in the case report was presented at the Equine Hospital at the University of Edinburgh for assessment of a suspected solar puncture. The clients signed a consent form to permit the clinical procedures and diagnostic imaging performed on the patient. The consent form also permitted the collection of anonymised postmortem samples for research purposes.

## Author Contributions

TF, LJM and RL were involved in clinical management of the case including review of diagnostic images. LRM prepared and interpreted the histopathological material. All authors were involved in the preparation of the manuscript.

## Conflict of Interest Statement

The authors declare that the research was conducted in the absence of any commercial or financial relationships that could be construed as a potential conflict of interest.
